# Novel reassortant of H1N1 swine influenza virus detected in pig population in Russia

**DOI:** 10.1080/22221751.2019.1673136

**Published:** 2019-10-11

**Authors:** Ivan Sobolev, Olga Kurskaya, Sergey Leonov, Marsel Kabilov, Tatyana Alikina, Alexander Alekseev, Yuriy Yushkov, Takehiko Saito, Yuko Uchida, Junki Mine, Alexander Shestopalov, Kirill Sharshov

**Affiliations:** a Department of Experimental Modeling and Pathogenesis of Infectious Diseases, Federal Research Center of Fundamental and Translational Medicine, Novosibirsk, Russia; b Siberian Federal Scientific Centre of Agro- BioTechnologies, Krasnoobsk, Russia; c Institute of Chemical Biology and Fundamental Medicine, Novosibirsk, Russia; d Division of Transboundary Animal Disease, National Institute of Animal Health, Tsukuba, Japan

**Keywords:** Swine, influenza, reassortant, phylogeny, H1N1

## Abstract

Pigs play an important role in interspecies transmission of the influenza virus, particularly as “mixing vessels” for reassortment. Two influenza A/H1N1 virus strains, A/swine/Siberia/1sw/2016 and A/swine/Siberia/4sw/2017, were isolated during a surveillance of pigs from private farms in Russia from 2016 to 2017. There was a 10% identity difference between the HA and NA nucleotide sequences of isolated strains and the most phylogenetically related sequences (human influenza viruses of 1980s). Simultaneously, genome segments encoding internal proteins were found to be phylogenetically related to the A/H1N1pdm09 influenza virus. In addition, two amino acids (129–130) were deleted in the HA of A/swine/Siberia/4sw/2017 compared to that of A/swine/Siberia/1sw/2016 HA.

## Introduction

Swine influenza is an acute respiratory disease of swine caused by the influenza A virus. Influenza A viruses (Orthomyxoviridae, Influenzavirus A) are isolated from a wide range of hosts, including birds, dogs, seals, horses, pigs, and humans [1]. Although influenza viruses rarely cross interspecies barriers, genome segments can overcome this barrier through a genetic reassortment process, or a genetic shift. Because avian influenza viruses and human influenza viruses are able to replicate in pigs, pigs play an important role in the interspecies transmission of the virus, as “mixing vessels” for the reassortment of viruses from different hosts [2]. The emergence of new influenza viruses that can overcome the interspecies barrier and human infections can occur as a result of this process.

Generally, pig to human transmission of the influenza virus of swine (IAV-S) occurs only rarely. However, sporadic cases of human infection caused by IAV-S have been reported. A case of human infection with the H1N1 IAV-S was officially documented and confirmed in 1958 [3,4]. There have been multiple reports of humans being infected with IAVs-S. For example, cases of human infection via H1N1v, H3N2v, and H1N2v viruses have been described in the United States [5–7]. Mostly, those infected with variant viruses, such as H1N1v, H3N2v, and H1N2v were children who had direct, or indirect, contact with pigs. Transmission of these variant viruses occurred between persons in close contact with each other and was, therefore, limited and not stable [8,9].

The virus, A/H1N1, which caused the influenza pandemic of 2009, is known to have crossed over to the human population from the pig population. This confirms the occurrence of viral genome reassortment in pigs and the subsequent danger of a novel virus being transmitted to humans. Thus, it is important to track influenza viruses which appear among pigs, particularly if these viruses differ from common variants.

Previously, IAV-S surveillance in the European part of Russia showed phylogenetic similarities between H1N1 strains collected in 2013–2014 and H1N1pdm09 [10]. In addition, except for those presented in this study, only 10 Russian IAV-S sequences (more than one segment represented) are available in the GISAID database. Of these, 7 were sequences of H1N1 IAV-S (presented only HA and NA), 1 was a sequence of H1N2 (complete genome except MP), 2 were H3N2 (complete genomes). Thus, the availability of data regarding genetic diversity and phylogenetic relationships of influenza viruses circulating in the pig population in Russia is reported to be inadequate. Private pig farms in Russia are dispersed over vast areas and are separated by considerable distances. This ensures the existence of many local pig populations harboring genetically different variants of the influenza virus. The current study investigated the causes of pig morbidity in a private farm, and detected variants of IAV-S with surface glycoproteins that are distinguishable from those of H1N1 IAVs-S reported previously.

## Materials and methods

### Sample collection

A total of 16 lung tissue samples were collected from fattened pigs aged 47–160 d in a private pig farm in Western Siberia, on the border of Ural region. Seven samples were obtained in March 2016 and nine in April 2017, during an outbreak of the porcine respiratory disease complex (PRDC). All pigs showed clinical symptoms as well as pathomorphological signs in lungs typical of Actinobacillus pleuropneumoniae infections (App). Actinobacillus pleuropneumoniae was isolated from all samples.

### Influenza virus detection and isolation

Virus isolation and identification were performed according to standard protocols at the BSL 3 Laboratory of Federal Research Centre of Fundamental and Translational Medicine. Lung samples were homogenized in 1 ml of Eagle's minimal essential medium containing the antibiotics, penicillin and gentamicin, and centrifuged at 4000 rpm for 5 min. The supernatant was collected and used for virus detection via real-time PCR and virus isolation in cell culture.

Samples were processed and analyzed for Influenza virus RNA via Real Time PCR using the “AmpliSens® Influenza virus A/B-FL” Influenza virus A and Influenza virus В RNA detection kit (InterLabService, Russia), according to the manufacturer's instructions.

Virus isolation was performed in Madin-Darby canine kidney (MDCK) cells by inoculating lung homogenates into cell monolayers. The supernatants of cultures showing cytopathic effects were harvested and tested via a hemagglutination (HA) test with 0.5% chicken red blood cells, and virus isolation was confirmed via RT–PCR. The isolated strains have been stored at −80°C in the depositarium of Federal Research Center of Fundamental and Translational Medicine.

### Antigenic analysis

Antigenic properties of the swine influenza viruses, A/swine/Siberia/1sw/2016 (H1N1) and A/swine/Siberia/4sw/2017 (H1N1), were characterized using a hemagglutination inhibition (HI) assay with mice polyclonal antisera against A/New Caledonia/22/99 (H1N1), A/Novosibirsk/653/2009 (H1N1), A/California/07/2009 (H1N1)pdm09, A/swine/Siberia/1sw/2016 (H1N1) and A/swine/Siberia/4sw/2017 (H1N1).

### Experimental infection of mice

All animal experiments were approved by the Ethics Committee of the Federal Research Centre of Fundamental and Translational Medicine. To evaluate the replication ability of A/swine/Siberia/1sw/2016 (H1N1) virus in mice, a group of 30, 6-week-old BALB/c mice were lightly anesthetized and intranasally inoculated with 106 tissue culture infective doses (TCID50) of the virus in 50 µl of cell culture supernatant. A group of negative control mice was inoculated intranasally with 50 µl of PBS. Animals were weighed and observed daily for 14 d post infection (p.i.). Three mice from the experimental group were euthanized on days 1, 3, 5, 7, and 10 p.i. and lung tissue samples were aseptically collected for virus titration. Each tissue sample was homogenized in 1 mL of Eagle's minimal essential medium containing the antibiotics, penicillin and gentamicin, and centrifuged at 4000 rpm for 5 min, and the supernatant was used to inoculate MDCK cells with an initial dilution of 1:10. Virus titres were calculated via the Kerber method.

On day 21 p.i., all animals were euthanized, blood samples were collected, and serum was obtained. Serum samples were tested via a HI assay for detection of antibodies to A/swine/Siberia/1sw/2016 (H1N1) virus.

### Sequencing

RNAs were isolated from cultured viral particles using a GeneJET viral DNA/RNA purification kit (Thermo Fisher Scientific) and treated with TURBO DNase (Thermo, Fisher Scientific). Up to 200 ng of RNA were used for the DNA libraries, which were prepared using a TruSeq RNA sample preparation kit version 2 (Illumina). Sequencing of the DNA libraries was conducted with a reagent kit, version 3 (600-cycle), on a MiSeq genome sequencer (Illumina) at SB RAS Genomics Core Facility (ICBFM SB RAS, Novosibirsk, Russia). Full-length genomes were assembled de novo with CLC Genomics Workbench version 9.0 (Qiagen).

Sequence accession numbers in GISAID (“global initiative on sharing avian flu data”) were as follows: EPI_ISL_231655 for A/swine/Siberia/1sw/2016; and EPI_ISL_314823 for A/swine/Siberia/4sw/2017.

### Sequence analysis

The swine influenza virus sequences being investigated were combined with sequences retrieved from the GISAID and IRD (Influenza Research Database) databases. For multiple alignments, a MUSCLE programme was used [11]. Comparative pairwise sequence alignment of 2 investigated strains was performed via BioEdit. Phylogenetic trees were built via MEGA 5 using Maximum Likelihood, utilizing the general time reversible (GTR) nucleotide substitution model. Bootstrap support values were generated using 500 rapid bootstrap replicates.

## Results

During the exacerbation of porcine respiratory disease complex in a private farm in March 2016 and in April 2017, lung tissue samples were collected from pigs showing clinical signs of the disease. A total of 16 samples were obtained from pigs aged 47–160 d. The total number of pigs monitored was 1,300. The farm was a farrow-to-finish operated with pigs that are bred and fattened in the farm. Vaccination against influenza was not conducted in this farm. All samples were positive for Actinobacillus pleuropneumoniae via ELISA.

Influenza A virus detection in lung sample homogenates was performed via RT–PCR using a commercial kit. Two samples were collected in March 2016 and two samples were collected in April 2017 from pigs aged 47–160 d, that were positive for the Influenza A virus. Following inoculation of MDCK cells with the samples, we isolated 2 Influenza viruses: A/swine/Siberia/1sw/2016 (H1N1) and A/swine/Siberia/4sw/2017 (H1N1), hereafter referred to as A/Sw2016 and A/Sw2017, respectively. Both viruses were isolated from pigs aged 57 d.

Antigenic analysis of A/Sw/2016 and A/Sw/2017 was performed using polyclonal mice antisera against different Influenza A (H1N1) viruses (Table 1). Both isolates did not demonstrate cross-reactivity with mice antisera against the 2009 pandemic influenza strain and pre-pandemic seasonal H1N1 influenza strains that circulated in the human population in 1999 and 2009. Antigenically, A/Sw/2016 demonstrated HI titres that were similar to those of A/Sw/2017 mice antiserum as well as to homologous antiserum. However, A/Sw/2017 showed a 4-fold reduction in HI titre to the serum against A/Sw/2016.

**Table 1.** Antigenic analysis of A/Sw/2016 and A/Sw/2017 in HI test. Assay was started from first dilution 1/10. The “5” value indicates the absence of cross-reactivity between virus and antisera (below the first dilution).

	A/New Caledonia/22/99 (H1N1)	A/Novosibirsk/653/2009 (H1N1)	A/California/07/2009 (H1N1)pdm09	A/Sw/2016	A/Sw/2017
A/NewCaledonia/22/99 (H1N1)	320	160	5	5	5
A/Novosibirsk/653/2009 (H1N1)	40	640	5	5	5
A/California/07/2009 (H1N1)pdm09	5	5	1280	5	5
A/Sw/2016	5	5	5	320	160
A/Sw/2017	5	5	5	160	640

### Experimental infection of mice

**Figure 1.**
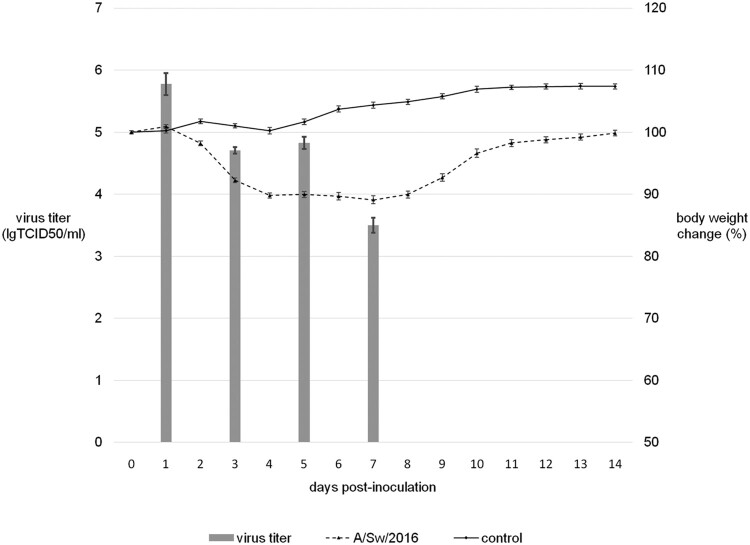
Experimental infection of Balb/c mice by A/Sw/2016 influenza virus.

The influenza virus, A/Sw/2016, was used to infect Balb/c mice experimentally. Intranasal inoculation of this isolate led to a productive infection, with virus replication in mice lungs during the first week p.i. (Figure 1). Although the infected mice did not die, all experimentally infected animals developed clinical symptoms such as weight loss, ruffled fur, hunched posture, and shivering. They began to lose weight on the 2nd d p.i. and maximum decrease in body weight, of up to 10% of initial body weight, was observed on 5th d p.i. All mice sera collected on the 21st d p.i. had HI antibodies to A/Sw/2016 (data not shown).

### Genetic analysis

The HA and NA nucleotide sequences of A/Sw2016 and A/Sw2017 strains were at least 10% different from the sequences presented in the databases, GenBank, GISAID and IRD. According to BLAST, HA nucleotide sequences of investigated strains shared 90% identity with the A/Memphis/7/1980 H1N1 strain, and other similar seasonal strains of the human influenza virus circulating in the world during the 70s and 80s. There were no HA sequences that were more than 90% similar to A/Sw2016 and A/Sw2017 in any database (GISAID, GenBank or IRD). A similar BLAST result was obtained for the NA gene sequences as follows: Russian strains were 90% identical to the strains of seasonal human influenza A/Memphis/49/1983 and A/Baylor/11515/1982; and gene segments coding internal proteins of the Russian strains were 97–99% identical to influenza virus A H1N1 pdm09 strains (Table 2).

**Table 2.** Nucleotide identity of near homologs in GISAID to the influenza A(H1N1) strains A/Sw2016 and A/Sw2017.

	A/Sw/2016		A/Sw/2017		A/Sw/2016 - A/Sw/2017
Segment	Name	% of identity	Name	% of identity	% of identity
PB2	A/St._Petersburg/204/2010	98,21	A/California/VRDL102/2009	97,46	98,90
	A/Arkhangelsk/CRIE-GNY/2009				
	A/Oslo/INS110/2009				
PB1	A/Australia/26/2009	98,20	A/Australia/26/2009	97,85	99,38
PA	A/Singapore/DMS913/2009	98,22	A/Wisconsin/629-S0254/2009	98,05	99,39
	A/Wisconsin/629-S0254/2009				
HA	A/Memphis/7/1980	90,48	A/Memphis/7/1980	89,79	98,77
NP	A/Florida/01/2010	98,26	A/Florida/01/2010	98,20	99,27
NA	A/Memphis/49/1983	90,54	A/Memphis/49/1983	90,30	99,28
	A/Baylor/11515/1982		A/Baylor/11515/1982		
MP	A/Russia/165/2009	99,08	A/Russia/165/2009	98,78	99,69
NS	A/Qingdao/F966/2010	98,57	A/Qingdao/F966/2010	98,33	99,28
	A/Russia/12/2009		A/Russia/12/2009		

In addition, a phylogenetic analysis was performed for genes encoding internal proteins (Supplementary Figs. 1–6). The genes of the internal proteins between the 2 investigated strains were similar and formed a separate phylogenetic group related to H1N1pdm09.

**Figure 2.**
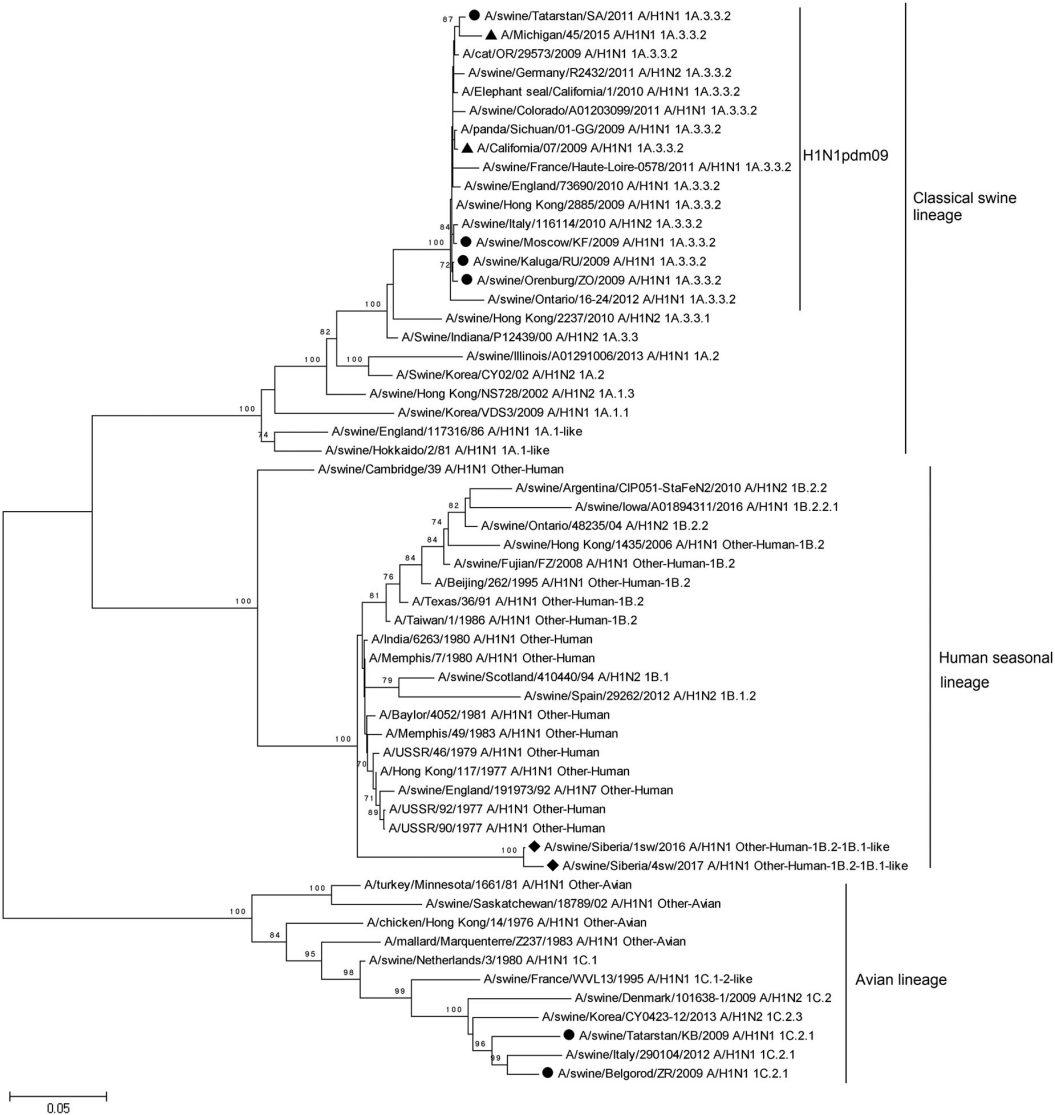
Phylogenetic analysis of HA-gene of A/Sw2016 and A/Sw2017 (rhombuses). Circles – other Russian swine H1N1 viruses. Triangles – H1N1pdm09 virus vaccine strains. Each sequence is classified according to a Phylogeny-Based Global Nomenclature System and Automated Annotation Tool for H1 Hemagglutinin Genes from Swine Influenza A Viruses.

**Figure 3.**
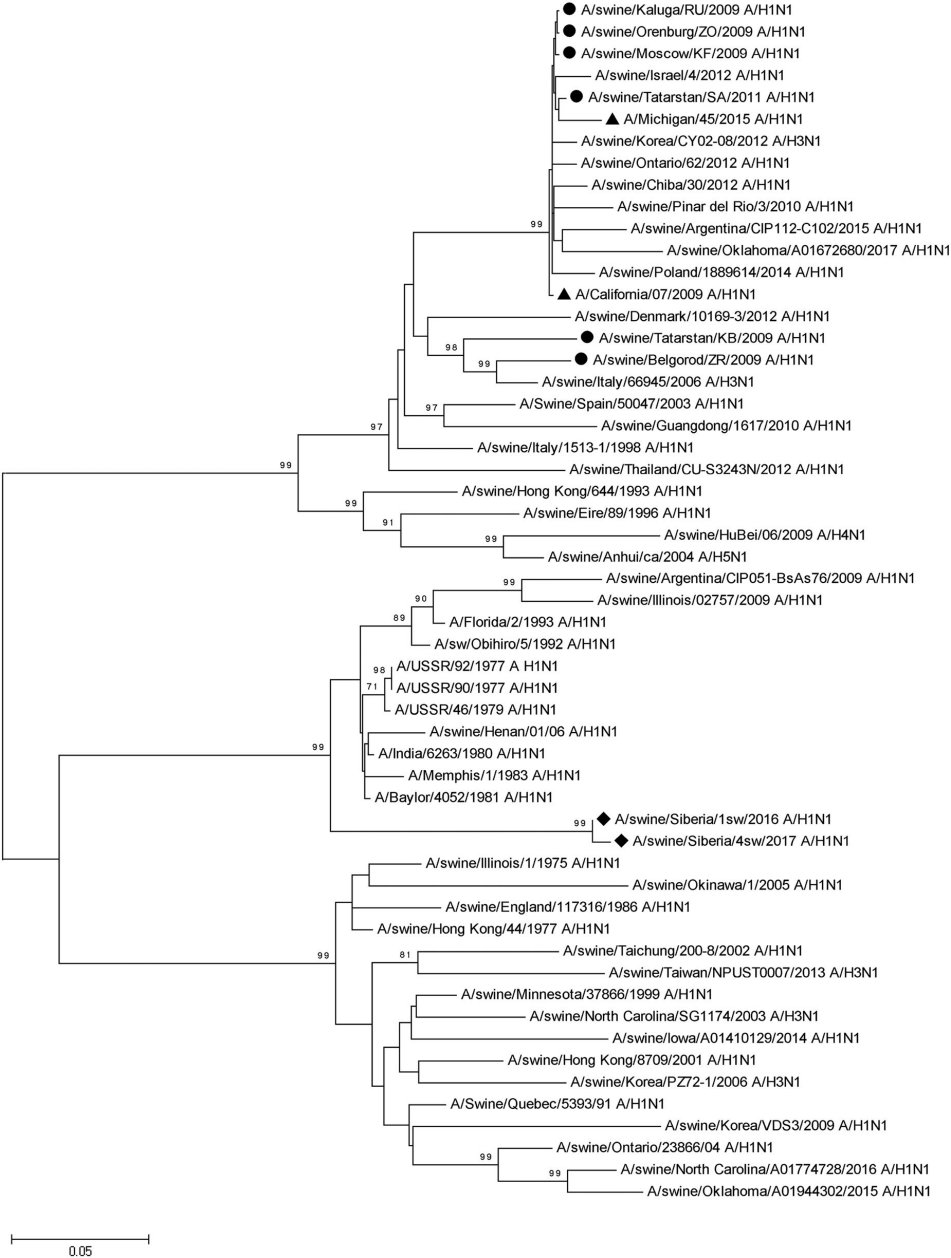
Phylogenetic analysis of NA-gene of A/Sw2016 and A/Sw2017 (rhombuses). Circles – other Russian swine H1N1 viruses. Triangles – H1N1pdm09 virus vaccine strains.

The BLAST results were confirmed by a phylogenetic analysis of the Russian strain nucleotide sequences (Figures 2 and 3). In general, swine influenza viruses with H1 are represented by the 2 subtypes, H1N1 and H1N2, although there are other possible variations (e.g. H1N7). These subtypes are divided into 3 large genetic groups: classic swine influenza [12–17], bird origin swine influenza [18–20], and human origin swine influenza [21–23]. Based on the phylogenetic differences of H1, there were several options for splitting the swine influenza virus into smaller genetic groups within the 3 main groups. The number and nomenclature of these groups were different between the swine influenza classifications in USA and Europe [24–26]. Therefore, OFFLU swine influenza experts published “A phylogeny-based global nomenclature system and automated annotation tool for H1 hemagglutinin genes from swine influenza A viruses,” which offered a resolution to the confusion associated with clade classification [27].

The HA of A/Sw2016 and A/Sw2017 strains form a separate genetic subgroup within the “Human seasonal influenza” clade according to the Swine H1 Clade classification which consists of isolates from humans and pigs (Figure 2). The subgroup of new Russian strains can be classified as other-Human-1B.2-1B.1-like. In addition to the human influenza strains of the 1970s and 1980s mentioned previously (human seasonal influenza lineage emerged when “Russian flu” appeared in 1977, including the human H1N1 strains from 1977 to 1980s), other-Human-1B.2-1B.1-like subgroup includes later strains isolated from pigs up to 2016, but based on a phylogenetic analysis and according to a matrix of pairwise genetic distances, these strains are not closely related to A/Sw2016 or A/Sw2017.

Strains of the swine influenza virus, previously isolated in Russia, were also included in the phylogenetic analysis. All these strains are phylogenetically different from HA of the new isolates and belong to other genetic lines. Strains A/swine/Tatarstan/KB/2009 and A/swine/Belgorod/ZR/2009 belong to the genetic subgroup 1C.2.1 of avian-like IAV-S. This group also includes strains isolated in Europe. A/swine/Moscow/KF/2009, A/swine/Orenburg/ZO/2009, A/swine/Kaluga/RU/2009, and A/swine/Tatarstan/SA/2011 belong to the genetic subgroup 1A.3.3.2 (H1N1pdm09-like influenza viruses) clade of classical swine lineage. In addition, IAVs-S isolated in 2013–2014 in the European part of Russia [10], were phylogenetically close to H1N1pdm09 influenza virus, and belonged to the 1A.3.3.2 genetic subgroup.

A phylogenetic analysis based on the nucleotide sequences of N1 NA also indicated differences between A/Sw2016 and A/Sw2017, and other strains represented in the genome databases (Figure 3). As in the case of HA, NA forms a separate genetic subgroup. Strains phylogenetically related (∼90% identity) to this subgroup were isolated from humans in the 70s and 80s. NA of the swine influenza virus strains isolated earlier in other regions of Russia and presented in databases, were phylogenetically different from NA of the strains investigated in our study. These strains are closely related to the swine influenza virus strains of the Eurasian genetic line (avian origin), including H1N1pdm09-like strains.

A/Sw2016 and A/Sw2017 were isolated at 1-year intervals from one pig population. A comparative analysis of amino acid sequences of internal proteins between investigated strains was conducted to evaluate the variability of this virus (Table 3). Strain A/California/07/2009 was added as a reference.

**Table 3.** Comparative analysis of the amino acid sequences of internal proteins between A/Sw2016 and A/Sw2017.

Protein	a.a. position	A/California/07/2009	A/Sw2016	A/Sw2017
PB2	54	R	K	K
	65	D	N	
	107	S	N	N
	194	Q	H	H
	225	G		S
	300	Q		H
	340	K	N	N
	511	V	I	I
PB1	182	T	I	I
	257	T	A	A
	386	K	R	R
	628	L	Q	Q
PB1-F2	5	L	*P*	*P*
	32	E	G	G
PA	19	K		R
	158	K	Q	Q
	212	R	L	L
	216	D	N	N
	224	*P*	S	S
	354	I		V
	369	A	V	V
	387	V	I	I
	535	H		Y
PA-X	19	K		R
	158	K	Q	Q
	207	L	S	S
	212	A	S	S
	225	A	V	V
	228	T	I	I
NP	34	G	D	
	38	R		K
	53	D	E	E
	100	V	I	I
	190	A	V	V
	313	V		I
	444	V	I	I
M2	24	D	N	N
	82	S		N
NS1	44	K	R	R
	98	M	L	L
	102	W		C
	123	I	V	V
	171	Y	Y	
	196	E		K
	197	N		D
NS2	14	M		V

The results demonstrate that, despite circulating within the same population of pigs, internal proteins of investigated strains are not only drift variants compared to the reference strain, but also differ from each other.

A comparative analysis of the HA and NA amino acid sequences of the A/Sw2016 and A/Sw2017 strains was also performed. Amino acid substitutions, I19 V, K45R, E120G, K139N, T190A, K450R and T488M (excluding the signal peptide), were identified between HA of these strains. In NA, amino acid substitutions G47E, T81I, N341D, and M418I were detected.

The investigated strains contained determinants of specificity for mammals, but not amino acid substitutions that increased virulence. It is known that H1N1 IAV receptor specificity largely depends on amino acid substitutions at positions 190 and 225 (according to H3 numbering without signal peptide) of HA. Hemagglutinin of the Russian IAV-S strains, A/swine/Siberia/1sw/2016 and A/swine/Siberia/4sw/2017, were characterized by the presence of D190 and D225 residues according to multiple alignment of corresponding sequences. The D190 + D225 combination determines IAV specificity to human α2–6 receptors [28,29]. PB2 sequences of A/swine/Siberia/1sw/2016 and A/swine/Siberia/4sw/2017 contained S590 and R591 residues that may facilitate virus replication in mammals [30]. PB1 sequences of investigated Russian IAV-S strains do not encode a PB1-F2, due to the presence of 3 premature stop codons at positions 12, 58, and 88. NS1 sequences contain the D92 residue instead of E92, which allows virus replication in the presence of IFN and induces high levels of virulence in pigs [31].

In addition, a deletion of 2 amino acids, 129–130 (146-147 with signal peptide), was detected in HA of A/Sw2017 compared to that of A/Sw2016 HA. Additional sequencing of necessary fragments of HA genome segments via the Sanger method was performed to confirm the deletion. Based on the NGS sequence, primers 5'-GTCCTACATTGCAGAAGCAC-3 ‘and 5'-GCGATAGATGGCCCTTTGAT-3’ were synthesized for amplification and sequencing of HA fragments containing a deletion. The results showed that A/Sw2016 does not contain a deletion of 129-130, but A/Sw2017 does.

## Discussion

Swine influenza viruses from 2 phylogenetic groups of classical swine lineage, 1C.2.1 avian influenza virus (Eurasian avian lineage) and 1A.3.3.2 (H1N1pdm09-like influenza virus), were isolated from pigs in Russia. Strains that were most recently identified by other research teams belonged to the group 1A.3.3.2. The current study reports the detection of novel reassortant variants of the influenza virus in a limited swine population of 1300. There was a 10% identity difference between the HA and NA nucleotide sequences of the isolated strains and the most phylogenetically related sequences belonging to the Human seasonal lineage (Clades 1B.1 and 1B.2). We used TempEst [32] to explore the degree and pattern of temporal signalling in the phylogenetic group of the human seasonal lineage IAV-S (1.B.1, 1.B.1.2, 1.B.2, 1.B.2.2, 1.B.2.2.1 and “other-human” clades according to the Phylogeny-Based Global Nomenclature System and Automated Annotation Tool for H1 Hemagglutinin Genes from Swine Influenza A Viruses.). The analysis showed a temporal signal with correlation coefficients 0.9694 and 0.9911 for HA and NA, respectively, of analyzed human-origin IAV-S (Supplementary Figs. 7-8). Such correlation coefficients indicate a positive correlation between genetic divergence and sample collection time. However, 40 years without “intermediate forms” have passed between the new Russian strains and the strains phylogenetically closest to them. The lack of data regarding novel swine influenza viruses in Russia may be due to insufficient surveillance, especially of private farms. Thus, it is possible that the newly discovered strains are evolutionary variants of previously unknown AIV-S circulating in local pig populations that have not been subjected to influenza virus surveillance.

Investigated strains are reassortants between classical swine lineage (1A.3.3.2, H1N1pdm09-like influenza) and Human seasonal lineage (1B.1–1B.2). All genome segments encoding internal proteins are genetically similar to the pandemic version of the H1N1pdm09 influenza virus, whereas HA and NA are not pandemic.

However, the appearance of reassortant influenza viruses in pig populations occurs regularly and therefore pigs are considered a potential reservoir for the transmission and reassortment of influenza viruses.

In our opinion, the main feature of the detected variants of the influenza virus is the significant (10%) difference between genome segments encoding HA and NA, and other sequences presented in the databases. Moreover, the most genetically similar (90% of identity) variants of HA and NA circulated among the human population during the period from 1977 to 1980. Genetic differences between the investigated strains from the actual pandemic variant pdm09 and those from the pre-pandemic seasonal strains of the influenza virus were confirmed by HI-assay. Monitoring and diagnostics have not been carried out in this pig population and region of Russia yet. Therefore, it is possible that this virus has been circulating locally for more than 30 years. Furthermore, these viruses were isolated in an area located far away from other locations in Russia where different swine viruses have been characterized and there are no movement of pigs between these different regions. This indicates the antigenic differences between the investigated Russian strains and the influenza viruses of H1N1pdm09, currently circulating in human and pig populations. Such differences create the risk of transmission of a new, genetically and antigenically different, variant of the influenza virus from pigs to humans.

In addition, the A/Sw2017 strain contains a deletion of two amino acids in HA. Deletion 129–130 was previously detected in HA of the A H1N2 influenza virus isolated from pigs in Italy [33,34], Canada, and the USA [28]. Ana Moreno et al., [34] reported that, at the time of publication (2013), there were no data regarding other strains carrying a deletion of 129-130, except the Italian strains. A later publication [35], reported that H1N2 strains carrying HA with a deletion of 129-130 were isolated from pigs during the period from 2012 to 2015. In addition, a deletion in position 130 was detected in swine influenza virus strains in the United States, Germany, and European countries (according to BLAST-analysis and multiple alignment of HA sequences).

Italian strains of the swine influenza virus carrying a deletion of 129-130 in HA belong to the European, human-like H1N2 SIVs [33] (clade 1B.1.2.2 of Human seasonal lineage). North American strains belonged to the classical swine lineage 1A.1.1. The H1N1 strains isolated from pigs in France (in particular A/swine/France/22-120067/2012) belonged to the human seasonal lineage (clade 1B.1.2.3). Thus, the 129-130 deletion was detected in swine influenza virus strains, belonging to different phylogenetic clades. This deletion was earlier detected in H1N2 subtype influenza viruses and its current detection in IAV-S, indicates that this mutation may have occurred due to adaptation of the H1 HA gene in the pig population.

Thus, a reassortant variant of the influenza virus with HA and NA of unknown origin was identified in a limited population of pigs. Moreover, one of the two investigated strains was found to contain a relatively rare deletion of amino acids 129-130, which was not characteristic of any particular phylogenetic clade. Lack of data on the origins and distribution of the detected influenza virus variant indicates that further investigation of viruses circulating in the investigated local pig population, as well as among pigs in other farms in Russia, may be required.

## Supplementary Material

Supplemental Material
