# Supplementary material for: Novel reassortant of H1N1 swine influenza virus detected in pig population in Russia
Source: Emerg Microbes Infect. 2019 Oct 11;8(1):1456–64. doi: 10.1080/22221751.2019.1673136 (PMC6818105; doi:10.1080/22221751.2019.1673136)
Supplement: Supplemental Material [file TEMI_A_1673136_SM4397.docx]

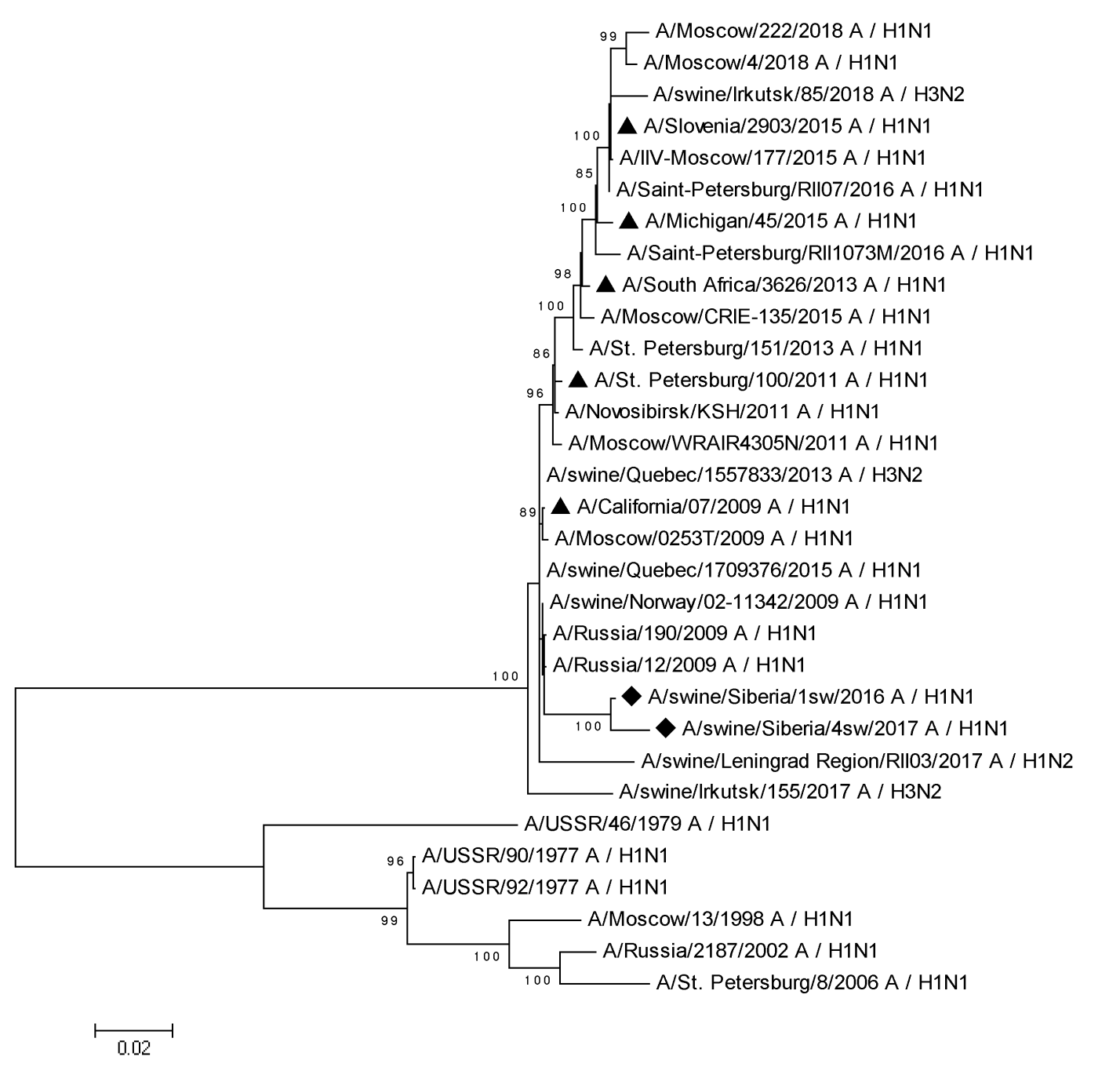
Supplementary Figure 1. Phylogenetic analysis of PB2 nucleotide sequence of A/Sw2016 and A/Sw2017 (rhombuses). Triangles – H1N1pdm09 virus vaccine and reference strains.


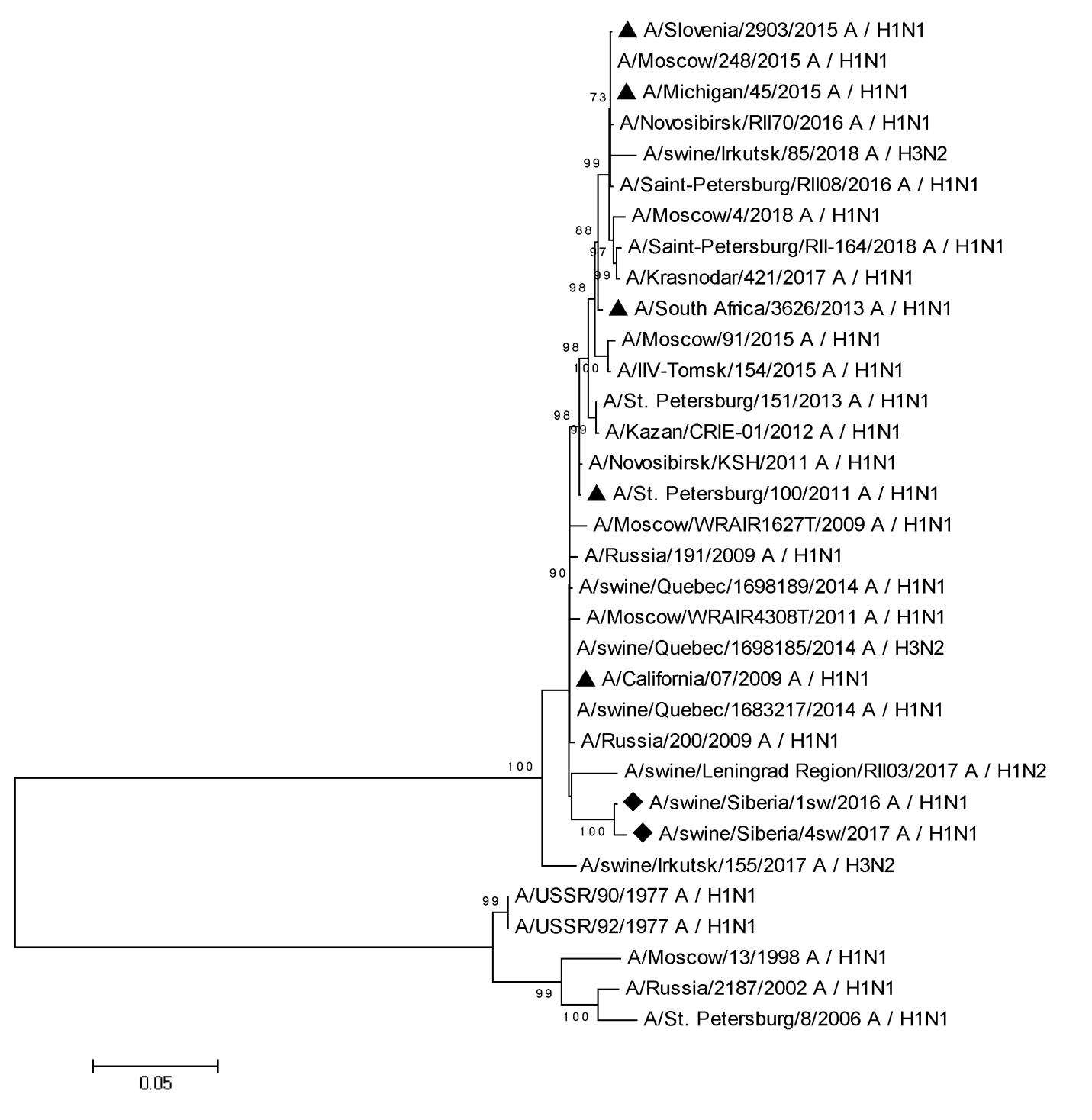
Supplementary Figure 2. Phylogenetic analysis of PB1 nucleotide sequence of A/Sw2016 and A/Sw2017 (rhombuses). Triangles – H1N1pdm09 virus vaccine and reference strains.


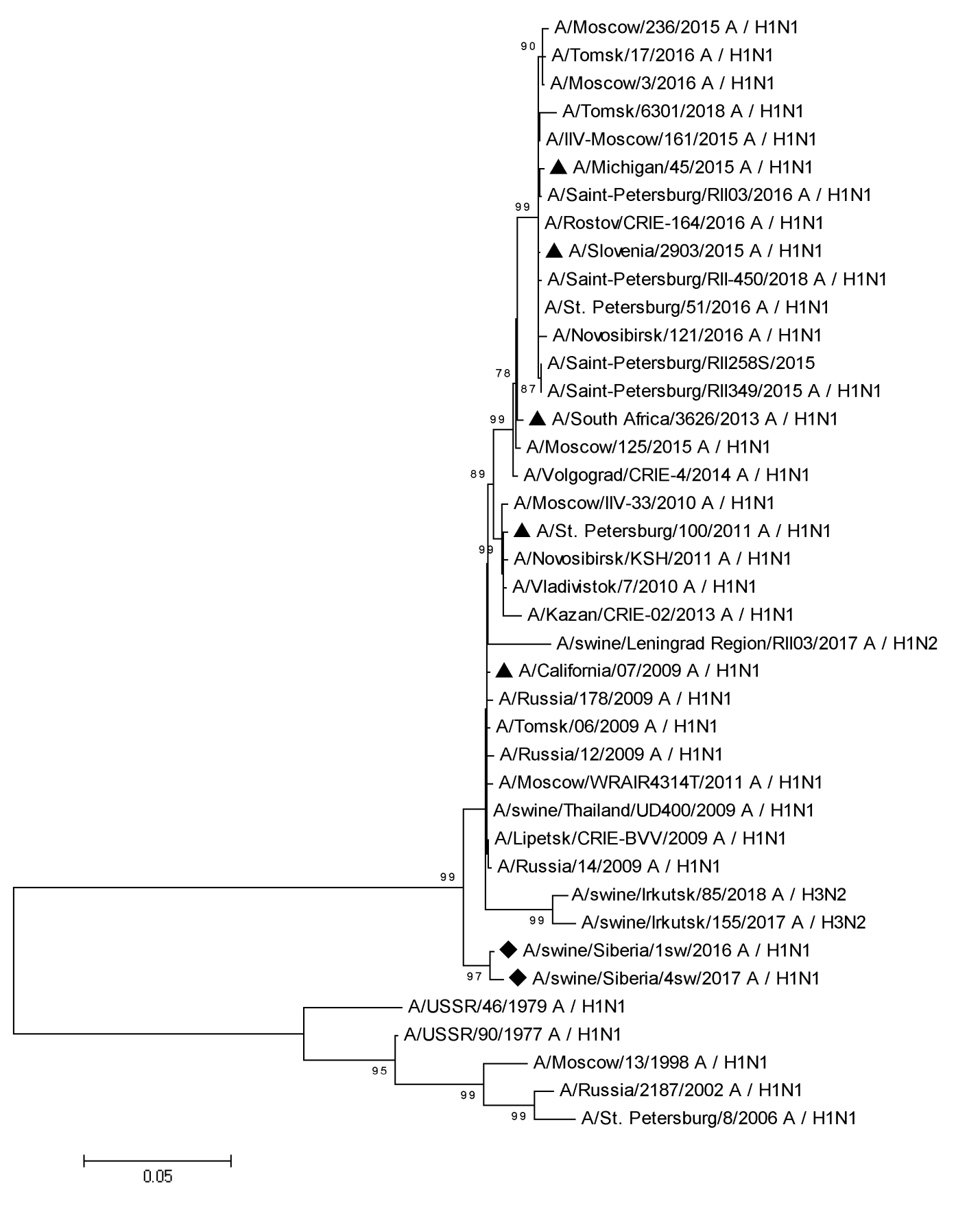
Supplementary Figure 3. Phylogenetic analysis of PA nucleotide sequence of A/Sw2016 and A/Sw2017 (rhombuses). Triangles – H1N1pdm09 virus vaccine and reference strains.


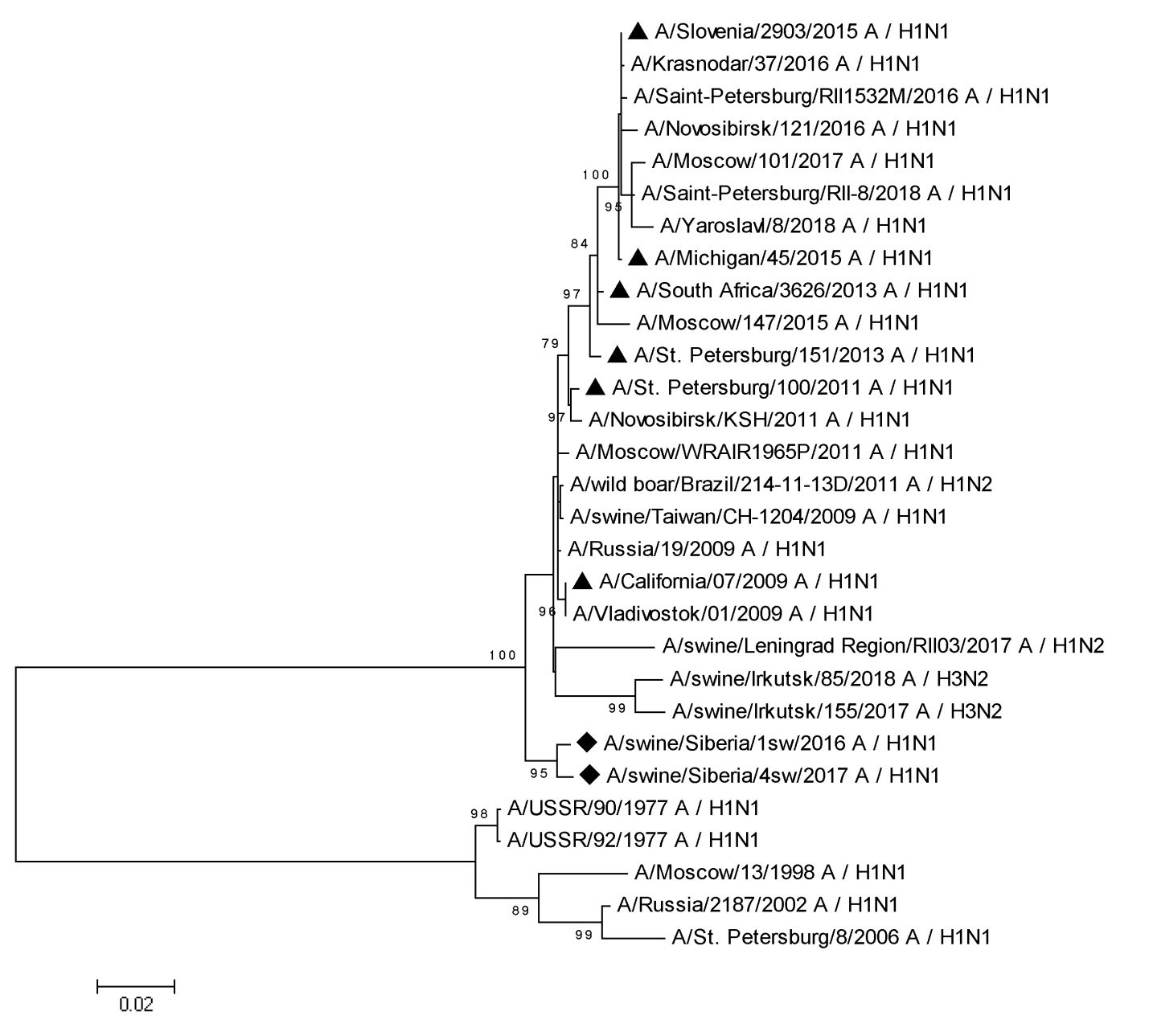
Supplementary Figure 4. Phylogenetic analysis of NP nucleotide sequence of A/Sw2016 and A/Sw2017 (rhombuses). Triangles – H1N1pdm09 virus vaccine and reference strains.


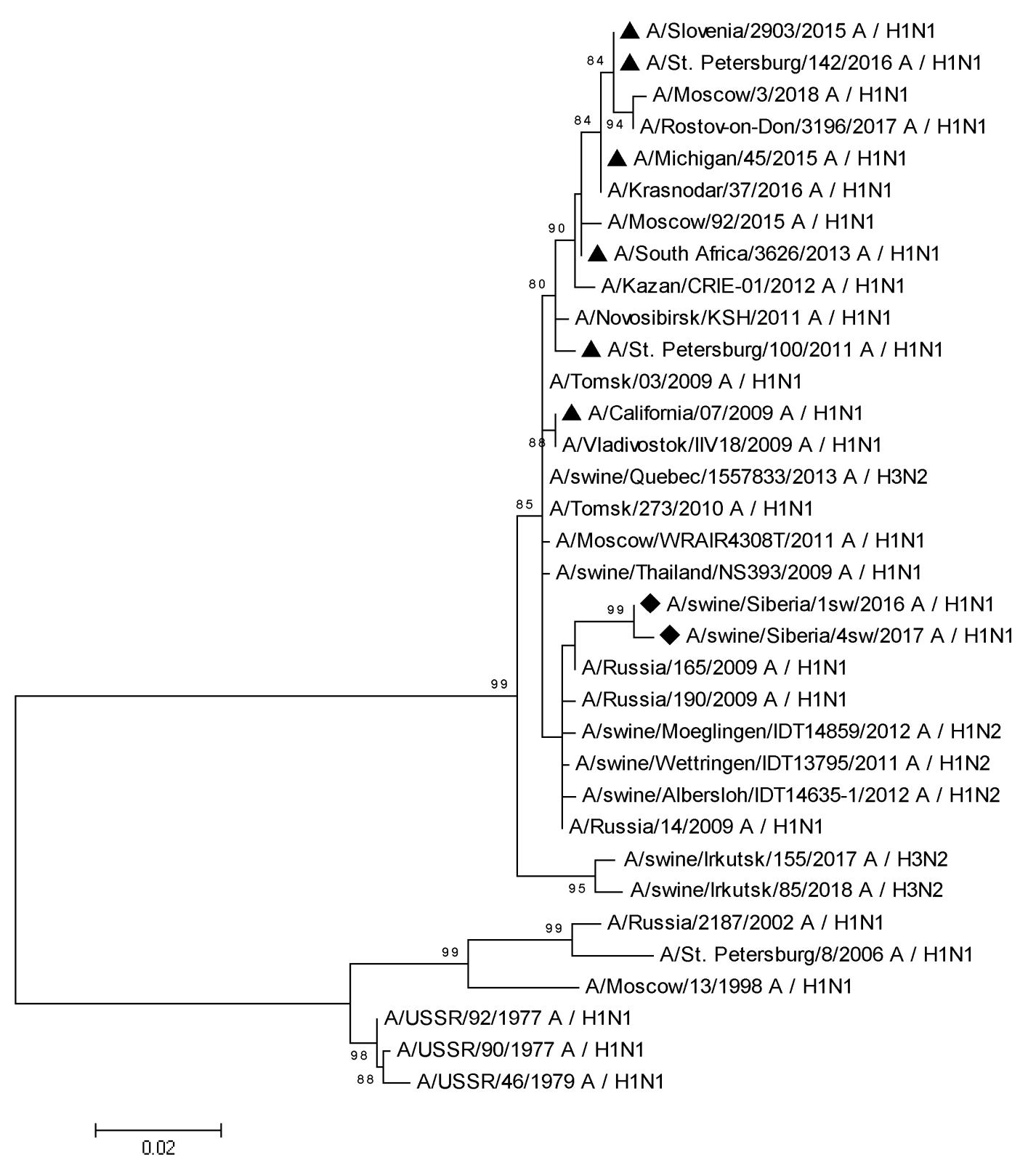
Supplementary Figure 5. Phylogenetic analysis of MP nucleotide sequence of A/Sw2016 and A/Sw2017 (rhombuses). Triangles – H1N1pdm09 virus vaccine and reference strains.


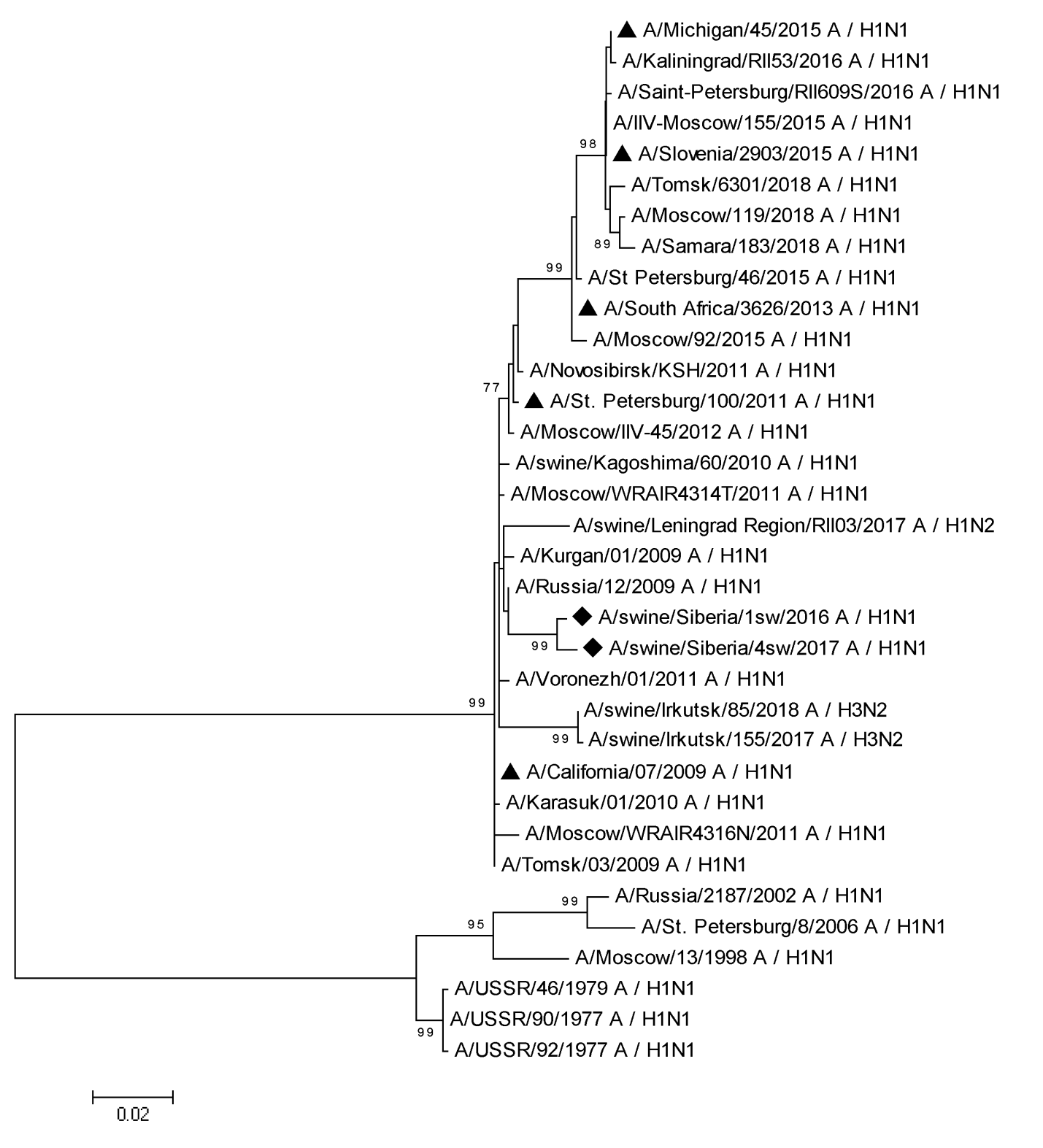
Supplementary Figure 6. Phylogenetic analysis of NS nucleotide sequence of A/Sw2016 and A/Sw2017 (rhombuses). Triangles – H1N1pdm09 virus vaccine and reference strains.


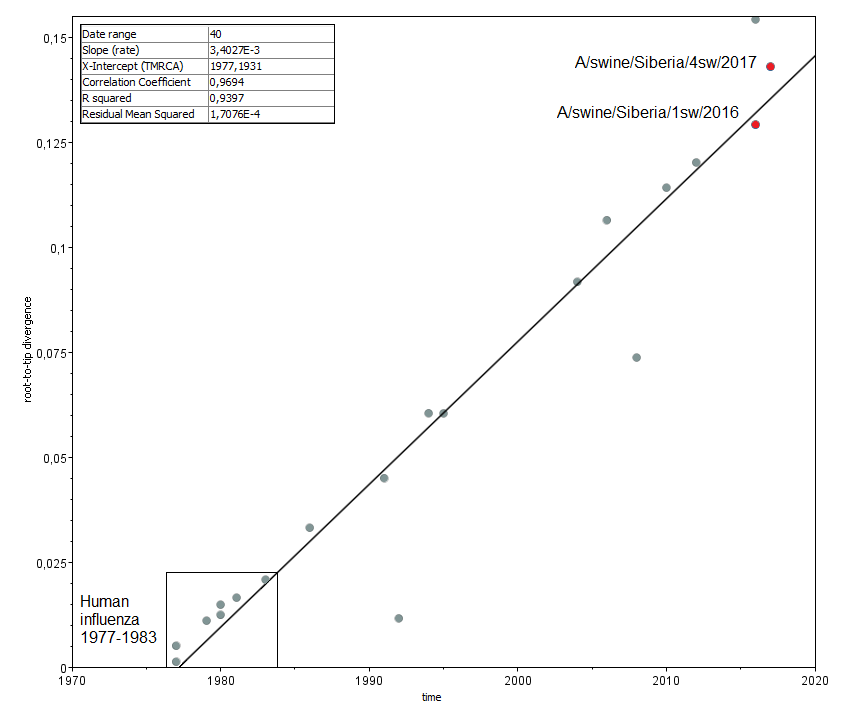
Supplementary Figure 7. Regression of root-to-tip genetic distance against samples collecting date for HA gene.


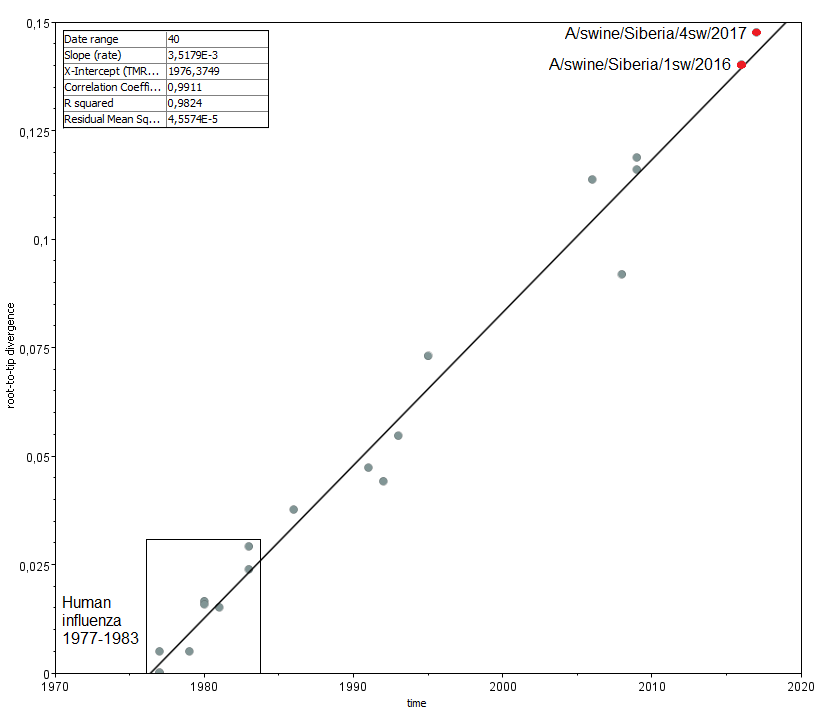
Supplementary Figure 8. Regression of root-to-tip genetic distance against samples collecting date for NA gene.
